# Effect of prior treatments on selinexor, bortezomib, and dexamethasone in previously treated multiple myeloma

**DOI:** 10.1186/s13045-021-01071-9

**Published:** 2021-04-13

**Authors:** Maria V. Mateos, Maria Gavriatopoulou, Thierry Facon, Holger W. Auner, Xavier Leleu, Roman Hájek, Meletios A. Dimopoulos, Sosana Delimpasi, Maryana Simonova, Ivan Špička, Ludĕk Pour, Iryna Kriachok, Halyna Pylypenko, Vadim Doronin, Ganna Usenko, Reuben Benjamin, Tuphan K. Dolai, Dinesh K. Sinha, Christopher P. Venner, Mamta Garg, Don A. Stevens, Hang Quach, Sundar Jagannath, Philippe Moreau, Moshe Levy, Ashraf Z. Badros, Larry D. Anderson, Nizar J. Bahlis, Michele Cavo, Yi Chai, Jacqueline Jeha, Melina Arazy, Jatin Shah, Sharon Shacham, Michael G. Kauffman, Paul G. Richardson, Sebastian Grosicki

**Affiliations:** 1 Hospital Universitario de Salamanca, Salamanca, Spain; 2 Alexandra Hospital, School of Medicine, National and Kapodistrian University of Athens, Athens, Greece; 3 CHU Lille Service Des Maladies du Sang, 59000 Lille, France; 4 Imperial College London, London, UK; 5 Department of Hematology, CHU La Miletrie and Inserm CIC 1402, Poitiers, France; 6 Department of Hematooncology, University Hospital Ostrava, Ostrava, Czech Republic; 7 National and Kapodistrian University of Athens, Athens, Greece; 8 General Hospital Evangelismos, Athens, Greece; 9 Institute of Blood Pathology and Transfusion Medicine of NAMS of Ukraine, Lviv, Ukraine; 10 Charles University and General Hospital, Prague, Czech Republic; 11 University Hospital Brno, Brno, Czech Republic; 12 National Cancer Institute, Kiev, Ukraine; 13 Cherkassy Regional Oncological Center, Cherkassy, Ukraine; 14 City Clinical Hospital #40, Moscow, Russian Federation; 15 City Clinical Hospital No. 4 of Dnipro City Council, Dnipro, Ukraine; 16 Kings College Hospital NHS Foundation Trust, London, UK; 17 Nil Ratan Sircar Medical College and Hospital, Kolkata, India; 18 State Cancer Institute, Indira Gandhi Institute of Medical Sciences, Patna, India; 19 Cross Cancer Institute, University of Alberta, Edmonton, AB Canada; 20 University Hospitals of Leicester NHS Trust, Leicester, UK; 21 Norton Cancer Institute, St. Matthews Campus, Louisville, KY USA; 22 St Vincent’s Hospital, University of Melbourne, Melbourne, VIC Australia; 23 Tisch Cancer Institute, Icahn School of Medicine at Mount Sinai, New York, NY USA; 24 Hotel-Dieu, University Hospital, Nantes, France; 25 Baylor University Medical Center, Dallas, TX USA; 26 Greenebaum Comprehensive Cancer Center, University of Maryland, Baltimore, MD USA; 27 Simmons Comprehensive Cancer Center, UT Southwestern Medical Center, Dallas, TX USA; 28 Charbonneau Cancer Research Institute, University of Calgary, Calgary, AB USA; 29 Seràgnoli Institute of Hematology, Bologna University School of Medicine, Bologna, Italy; 30 Karyopharm Therapeutics Inc, Newton, MA USA; 31 Dana Farber Cancer Institute, Boston, MA USA; 32 Medical University of Silesia, Katowice, Poland

**Keywords:** Selinexor, Exportin-1, Multiple myeloma, SINE compound

## Abstract

Therapeutic regimens for previously treated multiple myeloma (MM) may not provide prolonged disease control and are often complicated by significant adverse events, including peripheral neuropathy. In patients with previously treated MM in the Phase 3 BOSTON study, once weekly selinexor, once weekly bortezomib, and 40 mg dexamethasone (XVd) demonstrated a significantly longer median progression-free survival (PFS), higher response rates, deeper responses, a trend to improved survival, and reduced incidence and severity of bortezomib-induced peripheral neuropathy when compared with standard twice weekly bortezomib and 80 mg dexamethasone (Vd). The pre-specified analyses described here evaluated the influence of the number of prior lines of therapy, prior treatment with lenalidomide, prior proteasome inhibitor (PI) therapy, prior immunomodulatory drug therapy, and prior autologous stem cell transplant (ASCT) on the efficacy and safety of XVd compared with Vd. In this 1:1 randomized study, enrolled patients were assigned to receive once weekly oral selinexor (100 mg) with once weekly subcutaneous bortezomib (1.3 mg/m^2^) and 40 mg per week dexamethasone (XVd) versus standard twice weekly bortezomib and 80 mg per week dexamethasone (Vd). XVd significantly improved PFS, overall response rate, time-to-next-treatment, and showed reduced all grade and grade ≥ 2 peripheral neuropathy compared with Vd regardless of prior treatments, but the benefits of XVd over Vd were more pronounced in patients treated earlier in their disease course who had either received only one prior therapy, had never been treated with a PI, or had prior ASCT. Treatment with XVd improved outcomes as compared to Vd regardless of prior therapies as well as manageable and generally reversible adverse events. XVd was associated with clinical benefit and reduced peripheral neuropathy compared to standard Vd in previously treated MM. These results suggest that the once weekly XVd regimen may be optimally administered to patients earlier in their course of disease, as their first bortezomib-containing regimen, and in those relapsing after ASCT.

*Trial registration*: ClinicalTrials.gov (NCT03110562). Registered 12 April 2017. https://clinicaltrials.gov/ct2/show/NCT03110562.

**Supplementary Information:**

The online version contains supplementary material available at 10.1186/s13045-021-01071-9.

## To the Editor,

Therapeutic options have significantly advanced for patients with multiple myeloma (MM) including combination therapies employing complementary mechanisms or targeting mechanisms distinct from previous regimens [1, 2]. Selinexor is a first-in-class, orally-available, selective inhibitor of nuclear export (SINE) compound that has shown definitive activity with low dose dexamethasone in patients with triple class refractory MM in the STORM study [3] and synergistic activity with bortezomib and dexamethasone (XVd) in patients with 1–3 prior therapies in the BOSTON study [4]. Here we analyzed pre-specified subpopulations from the BOSTON study to determine the impact of prior lines of therapy and identify those who might optimally benefit from the XVd regimen.

Baseline characteristics were well balanced between treatment arms across subgroups (Additional file 1: Table S1). Median progression-free survival (PFS) was longer on XVd versus Vd in patients with 1 prior line (*P* = 0.0148) or 2–3 prior lines (*P* = 0.0295), lenalidomide-naïve (*P* = 0.0150) or lenalidomide-treated (*P* = 0.0177) patients, and PI-naïve patients (*P* = 0.0003), with a strong trend in PI-treated patients. Patients with IMiD-refractory MM had a significantly longer median PFS (*P* = 0.0051), as did patients with or without prior ASCT (*P* = 0.0074 and *P* = 0.0341). A post-hoc analysis showed a trend towards longer PFS with XVd in patients who received limited bortezomib induction prior to ASCT treatment (Table 1).

**Table 1:** Progression-free survival by subgroup

Patients (n, XVd vs Vd)	Median PFS, months (95% CI)		HR (95% CI)	*P* value
	XVd	Vd		
1 prior line (99 vs 99)	16.62 (13.24, NR)	10.68 (7.26, 16.39)	0.6295 (0.4133, 0.9586)	0.0148
2–3 prior lines (96 vs 108)	11.76 (7.39, NR)	9.43 (6.83, 9.69)	0.6949 (0.4760, 1.0147)	0.0295
Lenalidomide naïve (118 vs 130)	16.62 (12.98, NR)	10.61 (8.44, 15.41)	0.6619 (0.4548, 0.9634)	0.0150
Lenalidomide treated (77 vs 77)	9.59 (6.70, NR)	7.23 (4.93, 9.69)	0.6348 (0.4148, 0.9714)	0.0177
PI naïve (47 vs 48)	NR (NR, NR)	9.69 (8.44, NR)	0.2585 (0.1116, 0.5988)	0.0003
PI treated (148 vs 159)	11.73 (7.95, 15.21)	9.43 (7.06, 10.71)	0.7839 (0.5791, 1.0612)	0.0576
IMiD refractory (74 vs 86)	13.93 (6.70, NR)	8.44 (5.78, 9.56)	0.5752 (0.3753, 0.8816)	0.0051
Prior bortezomib only as induction for ASCT (37 vs 30)	13.14 (11.73, NR)	9.43 (5.75, NR)	0.5807 (0.2860, 1.1791)	0.0639
ASCT (76 vs 63)	16.56 (9.59, NR)	9.43 (5.91, 10.87)	0.5527 (0.3411, 0.8955)	0.0074
No ASCT (119 vs 144)	13.24 (10.18, NR)	9.56 (8.11, 13.60)	0.7239 (0.5111, 1.0252)	0.0341

*ASCT* autologous stem cell transplant, *CI* confidence interval, *IMiD* immunomodulatory drug, *NR* not reached, *ORR* overall response rate, *PFS* progression-free survival, *PI* proteasome inhibitor

**Fig. 1:**
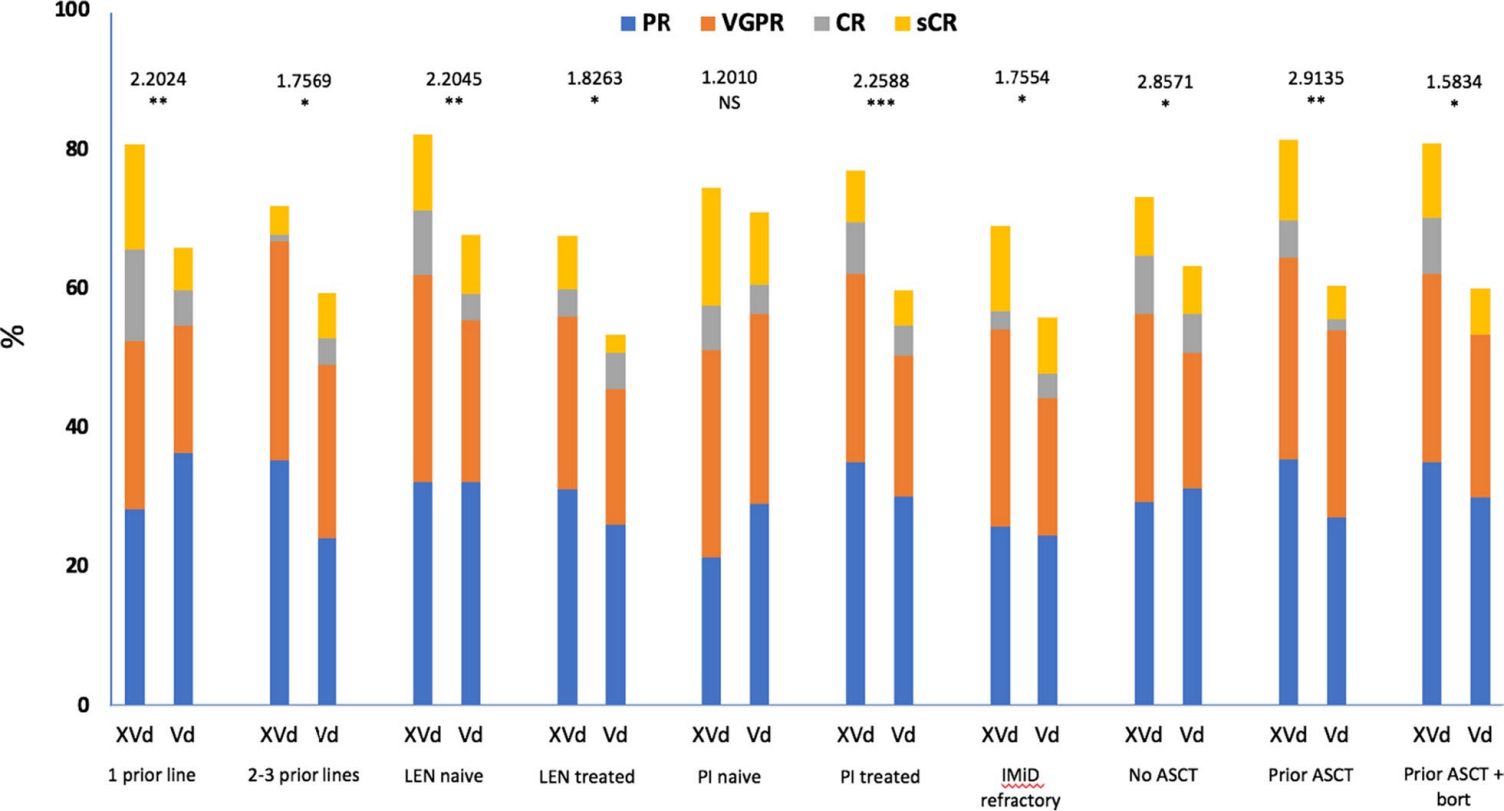
Depth of response by subgroup and treatment arm. The distribution of response pattern in subgroups based on number of prior lines, lenalidomide (LEN) or proteasome inhibitor (PI) treatment, IMiD refractoriness, and autologous stem cell transplant (ASCT). *Bort* bortezomib, *CR* complete response, *IMiD* immunomodulatory drug, *NS* not significant, *PR* partial response, *sCR* stringent complete response, *VGPR* very good partial response. Odds ratio and *P* value shown. *P < 0.05; **P < 0.01, ***P < 0.001

Treatment with XVd was associated with a significantly higher overall response rate including patients with 1 prior line, 2–3 prior lines, lenalidomide-naïve or treated, PI-naïve or treated, and prior ASCT (Fig. 1). Subgroups with 1 prior therapy, lenalidomide-naïve, and prior PI treatment had significantly higher rates of  ≥ VGPR (Additional file 1: Table S2). Median time-to-next-treatment was significantly improved with XVd versus Vd: 1 prior line, 2–3 prior lines, lenalidomide-naïve or treated, PI-naïve or treated, and prior ASCT. Across the entire study, overall survival (OS) trended in favor of XVd over Vd (HR, 0.84 [95% CI 0.57–1.23]; *P* = 0.19). The median OS for lenalidomide-naïve and PI-naïve patients was not reached, but favored XVd over Vd (HR, 0.76 [95% CI 0.45–1.29] *P* = 0.16 and HR 0.63 [95% CI 0.25–1.61], *P* = 0.16, respectively) (Additional file 1: Table S3).

Overall grade ≥ 3 adverse events (AEs) occurred more frequently with XVd and were generally well managed. Importantly, grade ≥ 2 peripheral neuropathy occurred significantly less frequently across all XVd subgroups. The incidence of serious AEs and drug discontinuation due to AEs trended higher with XVd (Additional file 1: Table S4). There was no clear trend regarding AEs leading to a fatal outcome, although the slight excess number of deaths with XVd in the PI-treated subgroup were restricted to India prior to the institution of increased monitoring, after which there were no additional deaths.

Our observations are particularly noteworthy as the once weekly XVd regimen utilizes ~ 40% less bortezomib and 25% less dexamethasone and requires ~ 37% fewer clinic visits for bortezomib injections than the standard Vd regimen. Despite the number of additional, subsequent therapies available to patients in this study, allowing patients on Vd with objective progressive disease to cross-over to a selinexor regimen, and the relatively short follow up, the results were accompanied by favorable trends on OS. Given its unique role in reactivating multiple tumor suppressor proteins and demonstrated synergy with PIs as well as other anti-MM drugs [5–9], these findings are consistent with the use of oral selinexor earlier in the MM treatment course. It is possible that some of the benefits of selinexor in those PI-treated patients may reflect the documented synergy between selinexor and PIs, even cells with marked PI refractoriness [5]. Moreover, benefits in duration and depth of response of XVd over Vd were most pronounced in patients who were PI-naïve, suggesting that selinexor could be an optimal partner for combining with *weekly* bortezomib as the first PI-containing MM regimen. Moreover, as daratumumab + lenalidomide/dexamethasone (DRd) is increasingly utilized in frontline MM treatment, the once weekly XVd regimen in second line could lead to a marked reduction in the development of prolonged or permanent bortezomib-associated neuropathy [10, 11]. Furthermore, the use of XVd following DRd allows for optimal mechanistic switching, thus preserving second generation agents (PIs, IMiDs and anti-CD38 mAbs) for subsequent lines of therapy where they may be more effective [1, 2, 12].

In conclusion, the earlier use of selinexor in treating MM may provide better, more durable outcomes with lower rates of peripheral neuropathy, using one of the simplest triplet regimens currently available for the treatment of patients with MM [4].

## Supplementary Information

**Additional file 1**. Supplementary material.

## Data Availability

Karyopharm Therapeutics agrees to share individual participant data that underlie the results reported in this article (after deidentification), including the study protocol and statistical analysis plan. Data availability will begin 9 months after publication and will be available 36 months after publication. To gain access, data requestors should submit a proposal to medicalinformation@karyopharm.com. Proposals will be reviewed by an independent review committee identified for this purpose.

## Abbreviations

ASCT: Autologous stem cell transplant
AE: Adverse event
CI: Confidence interval
DRd: Daratumumab plus lenalidomide/dexamethasone
HR: Hazard ratio
IMiDs: Immunomodulatory agents
MM: Multiple myeloma
NR: Not reached
ORR: Overall response rate
OS: Overall survival
PD: Progressive disease
PIs: Proteasome inhibitors
PFS: Progression-free survival
PN: Peripheral neuropathy
TTNT: Time-to-next-treatment
Vd: Bortezomib dexamethasone
VGPR: Very good partial response
XPO1: Exportin-1
XVd: Selinexor bortezomib dexamethasone
