# Supplementary material for: Effect of prior treatments on selinexor, bortezomib, and dexamethasone in previously treated multiple myeloma
Source: J Hematol Oncol. 2021 Apr 13;14:59. doi: 10.1186/s13045-021-01071-9 (PMC8045319; doi:10.1186/s13045-021-01071-9)
Supplement: Supplementary file 1 — Additional file 1. Supplementary material. [file 13045_2021_1071_MOESM1_ESM.docx]

**Effect of Prior Treatments on Selinexor, Bortezomib, and Dexamethasone**

**in Previously Treated Multiple Myeloma**

Maria V Mateos^1^, Maria Gavriatopoulou^2^, Thierry Facon^3^, Holger W. Auner^4^, Xavier Leleu^5^, Roman Hájek^6^,

Meletios A. Dimopoulos^7^, Sosana Delimpasi^8^, Maryana Simonova^9^, Ivan Špička^10^, Ludĕk Pour^11^, Iryna Kriachok^12^, Halyna Pylypenko^13^, Vadim Doronin^14^, Ganna Usenko^15^, Reuben Benjamin^16^, Tuphan K Dolai^17^, Dinesh K Sinha^18^, Christopher Venner^19^, Mamta Garg^20^, Don A Stevens^21^, Hang Quach^22^, Sundar Jagannath^23^, Philippe Moreau^24^,

Moshe Levy^25^, Ashraf Z. Badros^26^, Larry D. Anderson, Jr.^27^, Nizar J Bahlis^28^, Michele Cavo^29^, Yi Chai^30^,

Jacqueline Jeha^30^, Melina Arazy^30^, Jatin Shah^30^, Sharon Shacham^30^, Michael G. Kauffman^30^, Paul G. Richardson^31*^, Sebastian Grosicki^32*^

**Supplemental Material**

- **Methods**
- **Table S1. Baseline characteristics**
- **Table S2. Response rates according to prior treatment**
- **Table S3. Time to next treatment and overall survival according to prior treatment**
- **Table S4. Overall safety profile of XVd according to prior treatment**
- **Figure S1. Overall safety profile of XVd according to prior treatment**

**Methods**

**Study design and participants**

The BOSTON study has been described previously.^1^ The study enrolled patients ≥18 years of age with histologically confirmed MM with measurable disease per International Myeloma Working Group (IMWG) guidelines and had previously received 1-3 lines of anti-MM regimens and Eastern Cooperative Oncology Group (ECOG) performance status of 2 or less.
The study was approved and performed in accordance with the International Conference on Harmonization, the Guidelines for Good Clinical Practice, appropriate regulatory requirements, and with approval of institutional review boards at individual enrolling institutions. All patients provided written informed consent before study start.

The primary endpoint was PFS; secondary efficacy endpoints were ORR, OS, duration of response (DOR), time-to-next-treatment (TTNT), time to response (TTR) and grade ≥2 PN. All of the subpopulation analyses presented here were pre-specified except for the sensitivity analysis of patients with prior bortezomib used only as induction for ASCT.

**Assessments**

Patients underwent MM disease assessments at baseline and once every three weeks during each cycle. Overall response rate (ORR) was defined as CR, very good partial response (VGPR), and partial response (PR) based on the IMWG criteria ascertained by an IRC.^2^ All adverse events (AEs) and serious AEs (SAEs), regardless of relationship to study drug were recorded from first dose through at least 30 days after the last dose or until resolution of the AE.

**Statistical analysis**

A stratified log-rank test was used to compare the PFS, OS, DOR, TTNT, and TTR and the Cochran-Mantel-Haenszel (CMH) test for the ORR between treatment arms (XVd arm versus Vd arm). The strata included prior PI therapies, number of prior anti-MM regimens, and the Revised International Staging System (R-ISS) stage at study entry. The analyses presented here were all prespecified to compare the treatment arms for PFS, OS, TTNT for patients with 1 vs 2-3 prior therapies, prior lenalidomide, prior PI, and prior ASCT. A *post hoc* sensitivity analysis was also performed on subgroups of patients that were refractory to IMiDs and those that received bortezomib *only* in the context of ASCT induction.

**References**

1. Grosicki S, Simonova M, Picka I, et al. Once-weekly selinexor, bortezomib, and dexamethasone versus twice-weekly bortezomib and dexamethasone in patients with multiple myeloma (BOSTON): a randomised, open-label phase 3 trial. Lancet 2020;396:1563–73.

2. Kumar S, Paiva B, Anderson KC, et al. International Myeloma Working Group consensus criteria for response and minimal residual disease assessment in multiple myeloma. Lancet Oncol. 2016;17(8):e328–46.

**Table S1: Baseline Characteristics**

| **Prior treatments** | **1 prior line** | | **2 or 3 prior lines** | | **Lenalidomide naïve** | | **Lenalidomide treated** | | **PI naïve** | | **PI treated** | |  |
| --- | --- | --- | --- | --- | --- | --- | --- | --- | --- | --- | --- | --- | --- |
|  | **XVd**  **n=99** | **Vd**  **n=99** | **XVd**  **n=96** | **Vd**  **n=108** | **XVd**  **n=118** | **Vd**  **n=130** | **XVd**  **n=77** | **Vd**  **n=77** | **XVd**  **n=47** | **Vd**  **n=48** | **XVd**  **n=148** | **Vd**  **n=159** |  |
| **Age (years), median (range)** | 67.0 (45, 87) | 69.0 (44, 90) | 65.5 (40, 84) | 65.0 (38, 85) | 66.0 (45, 84) | 68.0 (38, 90) | 65.0 (40, 87) | 66.0 (45, 85) | 68.0 (45, 87) | 68.0 (44, 84) | 65.0 (40, 84) | 67.0 (38, 90) |  |
| **Male sex, n (%)** | 55 (55.6) | 53 (53.5) | 60 (62.5) | 62 (57.4) | 62 (52.5) | 75 (57.7) | 53 (68.8) | 40 (51.9) | 26 (55.3) | 27 (56.3) | 89 (60.1) | 88 (55.3) |  |
| **ECOG performance-status, n (%)** |  |  |  |  |  |  |  |  |  |  |  |  |  |
| 0 | 39 (39.4) | 38 (38.4) | 30 (31.3) | 39 (36.1) | 36 (30.5) | 46 (35.4) | 33 (42.9) | 31 (40.3) | 17 (35.4) | 32 (33.7) | 54 (36.5) | 60 (37.7) |  |
| 1 | 52 (52.5) | 55 (55.6) | 54 (56.3) | 59 (54.6) | 70 (59.3) | 73 (56.2) | 36 (46.8) | 41 (53.2) | 23 (47.9) | 49 (51.6) | 80 (54.1) | 91 (57.2) |  |
| 2 | 8 (8.1) | 6 (6.1) | 12 (12.5) | 10 (9.3) | 12 (10.2) | 11 (8.5) | 8 (10.4) | 5 (6.5) | 8 (16.7) | 14 (14.7) | 14 (9.5) | 8 (5.0) |  |
| **Frail status, n (%)** | 35 (35.4) | 28 (28.3) | 31 (32.3) | 36 (33.3) | 41 (34.7) | 39 (30.0) | 25 (32.5) | 25 (32.5) | 19 (40.4) | 22 (45.8) | 47 (31.8) | 42 (26.4) |  |
| **Number of prior therapies, median (range)** | 1 (1,1) | 1 (1,1) | 2.3 (2,3) | 2.3 (2,3) | 1 (1,3) | 1 (1,3) | 2 (1,3) | 2 (1,3) | 1 (1,3) | 1 (1,3) | 2 (1,3) | 2 (1,3) |  |
| **Prior ASCT, n (%)** | 39 (39.4) | 23 (23.2) | 37 (38.5) | 40 (37.0) | 43 (36.4) | 31 (23.8) | 33 (42.9) | 32 (41.6) | 13 (27.7) | 10 (20.8)) | 63 (42.6) | 53 (33.3) |  |
| **Previous therapy, n (%)** |  |  |  |  |  |  |  |  |  |  |  |  |  |
| Bortezomib | 64 (64.6) | 65 (65.7) | 70 (72.9) | 80 (74.1) | 81 (68.6) | 91 (70.0) | 53 (68.8) | 54 (70.1) | 0 (0) | 0 (0) | 134 (90.5) | 145 (91.2) |  |
| Carfilzomib | 7 (7.1) | 8 (8.1) | 13 (13.5) | 13 (12.0) | 8 (6.8) | 9 (6.9) | 12 (15.6) | 12 (15.6) | 0 (0) | 0 (0) | 20 (13.5) | 21 (13.2) |  |
| Ixazomib | 1 (1.0) | 1 (1.0) | 5 (5.2) | 2 (1.9) | 2 (1.7) | 2 (1.5) | 4 (5.2) | 1 (1.3) | 0 (0) | 0 (0) | 6 (4.1) | 3 (1.9) |  |
| Daratumumab | 3 (3.0) | 3 (3.0) | 8 (8.3) | 3 (2.8) | 4 (3.4) | 2 (1.5) | 7 (9.1) | 4 (5.2) | 0 (0) | 1 (2.1) | 11 (7.4) | 5 (3.1) |  |
| Lenalidomide | 23 (23.2) | 20 (20.2) | 54 (56.3) | 57 (52.8) | 0 (0) | 0 (0) | 77 (100.0) | 77 (100.0) | 18 (38.3) | 17 (35.4) | 59 (39.9) | 60 (37.7) |  |
| Pomalidomide | 0 | 0 | 7 (6.5) | 18 (8.8) | 1 (0.8) | 4 (3.1) | 10 (13.0) | 3 (3.9) | 1 (2.1) | 0 (0) | 10 (6.8) | 7 (4.4) |  |
| **Prior treatments** | **IMiD refractory** | | **Prior bortezomib only** **as induction for ASCT** | | **ASCT** | | **No ASCT** | |  | | | | |
|  | **XVd**  **n=74** | **Vd**  **n= 86** | **XVd**  **n=37** | **Vd**  **n= 30** | **XVd**  **n=119** | **Vd**  **n= 63** | **XVd**  **n=119** | **Vd**  **n= 144** |  |  |  |  |  |
| **Age (years), median (range)** | 63.0(40, 87) | 66.0(38,85) | 62.0 (43, 72) | 64.0 (51, 75) | 63.0 (40, 78) | 63.0 (43,76) | 69.0 (42,87) | 70.0 (38,90) |  | | | | |
| **Male sex, n (%)** | 46 (62.2) | 46 (53.5) | 22 (59.5) | 20 (66.7) | 47 (61.8) | 39 (61.9) | 68 (57.1) | 76 (52.8) |  |  |  |  |  |
| **ECOG performance-status, n (%)** |  |  |  |  |  |  |  |  |  |  |  |  |  |
| 0 | 30 (40.5) | 33 (38.4) | 17 (45.9) | 14 (46.7) | 34 (44.7) | 33 (52.4) | 35 (29.4) | 44 (30.6) |  |  |  |  |  |
| 1 | 39 (52.7) | 44 (51.2) | 19 (51.4) | 16 (53.3) | 39 (51.3) | 30 (47.6) | 67 (56.3) | 84 (58.3) |  |  |  |  |  |
| 2 | 5 (6.8) | 9 (10.5) | 1 (2.7) | 0 | 3 (3.9) | 0 | 17 (14.3) | 16 (11.1) |  |  |  |  |  |
| **Frail status, n (%)** | 7 (18.9) | 2 (6.7) | 7 (18.9) | 2 (6.7) | 12 (15.8) | 4 (6.3) | 54 (45.4) | 60 (41.7) |  |  |  |  |  |
| **Number of prior therapies, median (range)** | 2 (1, 3) | 2 (1, 3) | 1 (1, 3) | 1 (1, 3) | 1(1, 3) | 2(1, 3) | 1(1, 3) | 1(1, 3) |  |  |  |  |  |
| **Prior ASCT, n (%)** | 27 (36.5) | 30 (34.9) | 37 (100.0) | 30 (100.0) | 76 (100) | 63 (100) | 0 | 0 |  |  |  |  |  |
| **Previous therapy, n (%)** |  |  |  |  |  |  |  |  |  |  |  |  |  |
| Bortezomib | 50 (67.6) | 56 (65.1) | 37 (100.0) | 30 (100.0) | 59 (77.6) | 49 (77.8) | 75 (63.0) | 96 (66.7) |  |  |  |  |  |
| Carfilzomib | 9 (12.2) | 8 (9.3) | 1 (2.7) | 1 (3.3) | 7 (9.2) | 6 (9.5) | 13 (10.9) | 15 (10.4) |  |  |  |  |  |
| Ixazomib | 2 (2.7) | 1 (1.2) | 0 | 1 (3.3) | 1 (1.3) | 2 (3.2) | 5 (4.2) | 1 (0.7) |  |  |  |  |  |
| Daratumumab | 7 (9.5) | 3 (3.5) | 0 | 0 | 3 (3.9) | 2 (3.2) | 8 (6.7) | 4 (2.8) |  |  |  |  |  |
| Lenalidomide | 57 (77.0) | 58 (67.4) | 15 (40.5) | 14 (46.7) | 33 (43.4) | 32 (50.8) | 44 (37.0) | 45 (31.3) |  |  |  |  |  |
| Pomalidomide | 10 (13.5) | 7 (8.1) | 0 | 2 (6.7) | 1 (1.3) | 4 (6.3) | 10 (8.4) | 3 (2.1) |  |  |  |  |  |

ASCT, autologous stem cell transplant; ECOG, Eastern Cooperative Oncology Group; IMiD, immunomodulatory drug; PI, proteasome inhibitor

**Table S2. Response rates according to prior treatment**

| **Patients**  **(n, XVd vs Vd)** | **ORR, % (95% CI)** | | | | **≥VGPR, % (95% CI)** | | | |
| --- | --- | --- | --- | --- | --- | --- | --- | --- |
|  | XVd | Vd | OR | *P value* | XVd | Vd | OR | *P value* |
| **1 prior line (99 vs 99)** | 80.8  (71.7, 88.0) | 65.7  (55.4, 74.9) | 2.2024  (1.1500, 4.2181) | 0.0082 | 52.5  (42.2, 62.7) | 29.3  (20.6, 39.3) | 2.6706  (1.4869, 4.7966) | 0.0005 |
| **2-3 prior lines (96 vs 108)** | 71.9  (61.8, 80.6) | 59.3  (49.4, 68.6) | 1.7569  (0.9763, 3.1619) | 0.0298 | 36.5  (26.9, 46.9) | 35.2  (26.2, 45.0) | 1.0569  (0.5957, 1.8752) | 0.4251 |
| **Lenalidomide naïve (118 vs 130)** | 82.2  (74.1, 88.6) | 67.7  (58.9, 75.6) | 2.2045  (1.2123, 4.0091) | 0.0044 | 50.0  (40.7, 59.3) | 35.4  (27.2, 44.2) | 1.8261  (1.0972, 3.0391) | 0.0101 |
| **Lenalidomide treated (77 vs 77)** | 67.5  (55.9, 77.8) | 53.2  (41.5, 64.7) | 1.8263  (0.9495, 3.5130) | 0.0354 | 36.4  (25.7, 48.1) | 27.3  (17.7, 38.6) | 1.5238  (0.7693, 3.0182) | 0.1137 |
| **PI naïve (47 vs 48)** | 74.5 (59.7, 86.1) | 70.8 (55.9, 83.0) | 1.2010  (0.4863, 2.9658) | 0.3464 | 53.2  (38.1, 67.9) | 41.7  (27.6, 56.8) | 1.5909  (0.7071, 3.5794) | 0.1316 |
| **PI treated (148 vs 159)** | 77.0 (69.4, 83.5) | 59.7 (51.7, 67.4) | 2.2588  (1.3740, 3.7135) | 0.0006 | 41.9  (33.8, 50.3) | 29.6  (22.6, 37.3) | 1.7180  (1.0717, 2.7539) | 0.0121 |
| **IMiD refractory (74 vs 86)** | 68.9  (57.1, 79.2) | 55.8  (44.7, 66.5) | 1.7554  (0.9157, 3.3651) | 0.0449 | 43.2  (31.8, 55.3) | 31.4  (21.8, 42.3) | 1.6649  (0.8716, 3.1803) | 0.0613 |
| **Prior bortezomib only as induction for ASCT (37 vs 30)** | 81.1  (64.8, 92.0) | 60.0  (40.6, 77.3) | 2.8571  (0.9509, 8.5848) | 0.0294 | 45.9  (29.6, 63.1) | 30.0  (14.7, 49.4) | 1.9833  (0.7196, 5.4662) | 0.0931 |
| **ASCT (76 vs 63)** | 81.6  (71.0, 89.5) | 60.3  (47.2, 72.4) | 2.9135  (1.3506, 6.2852) | 0.0028 | 46.1  (34.5, 57.9) | 33.3  (22.0, 46.3) | 1.7073  (0.8553, 3.4082) | 0.0647 |
| **No ASCT (119 vs 144)** | 73.1  (64.2, 80.8) | 63.2  (54.8, 71.1) | 1.5834  (0.9338, 2.6851) | 0.0438 | 43.7  (34.6, 53.1) | 31.9  (24.4, 40.2) | 1.6535  (0.9990, 2.7368) | 0.0251 |

ASCT, autologous stem cell transplant; CI, confidence interval; IMiD, immunomodulatory drug; NR, not reached; ORR, overall response rate; PI, proteasome inhibitor; VGPR, very good partial response

**Table S3. Time to next treatment and overall survival according to prior treatment**

| **Patients (n, XVd vs Vd)** | **Median TTNT, months**  **(95% CI)** | | **Median OS, months**  **(95% CI)** | |
| --- | --- | --- | --- | --- |
|  | XVd | Vd | XVd | Vd |
| **1 prior line (99 vs 99)** | NR  (16.13, NR) | 12.88  (9.82,16.23) | NR  (NR, NR) | 24.97  (21.22, NR) |
| **2-3 prior lines (96 vs 108)** | 14.03  (11.50, NR) | 10.81  (8.54,13.63) | NR  (20.44, NR) | NR  (24.84, NR) |
| **Lenalidomide naïve (118 vs 130)** | 19.12  (14.03, NR) | 13.14  (10.61,17.05) | NR  (NR, NR) | NR  (24.97, NR) |
| **Lenalidomide treated (77 vs 77)** | 13.93  (8.08, NR) | 8.80  (6.47, 11.73) | NR  (16.92, NR) | 23.49  (23.49, NR) |
| **PI naïve (47 vs 48)** | NR  (NR, NR) | 10.84  (10.15, NR) | NR  (21.39, NR) | NR  (23.49, NR) |
| **PI treated (148 vs 159)** | 14.03  (9.99,18.23) | 10.81  (8.71, 13.40) | NR  (NR, NR) | 24.97  (21.22, NR) |
| **IMiD refractory (74 vs 86)** | 14.78  (8.08, NR) | 10.18  (7.23, 12.09) | 21.39  (20.44, NR) | 24.84  (23.49, NR) |
| **Prior bortezomib only as induction for ASCT (37 vs 30)** | 11.93  (8.08, NR) | 9.18  (5.91, NR) | NR  (20.44, NR) | 21.22  (16.00, NR) |
| **ASCT (76 vs 63)** | 14.13  (9.99, NR) | 9.95  (7.16, 13.40) | NR  (NR, NR) | 24.84  (21.22, NR) |
| **No ASCT (119 vs 144)** | 16.92  (14.03, NR) | 12.45  (9.92, 15.34) | NR  (21.39, NR) | 24.97  (23.49, NR) |

ASCT, autologous stem cell transplant; CI, confidence interval; IMiD, immunomodulatory drug; NR, not reached; OS, overall survival; PI, proteasome inhibitor; TTNT, time to next treatment

**Table S4. Overall safety profile of XVd according to prior treatment**

|  | **1 prior line** | | **2 or 3 prior lines** | | **Lenalidomide naïve** | | **Lenalidomide treated** | | **PI naïve** | | **PI treated** | |  |
| --- | --- | --- | --- | --- | --- | --- | --- | --- | --- | --- | --- | --- | --- |
|  | **XVd**  **n=99**  **n (%)** | **Vd**  **n=98**  **n (%)** | **XVd**  **n=96**  **n (%)** | **Vd**  **n=106**  **n (%)** | **XVd**  **n=118**  **n (%)** | **Vd**  **n=129**  **n (%)** | **XVd**  **n=77**  **n (%)** | **Vd**  **n=75**  **n (%)** | **XVd**  **n=47**  **n (%)** | **Vd**  **n=48**  **n (%)** | **XVd**  **n=148**  **n (%)** | **Vd**  **n=156**  **n (%)** |  |
| **Treatment-Emergent Adverse Event** | 98 (99.0) | 96 (98.0) | 96 (100.0) | 102 (96.2) | 118 (100.0) | 126 (97.7) | 76 (98.7) | 72 (96.0) | 46 (97.9) | 46 (95.8) | 148 (100.0) | 152 (97.4) |  |
| **Grade 3/4 TEAE** | 76 (76.8) | 55 (56.1) | 78 (81.3) | 59 (55.7) | 90 (76.3) | 71 (55.0) | 64 (83.1) | 43 (57.3) | 36 (76.6) | 27 (56.3) | 118 (79.7) | 87 (55.8) |  |
| **Grade 4 TEAE** | 15 (15.2) | 13 (13.3) | 19 (19.8) | 9  (8.5) | 22 (18.6) | 14 (10.9) | 12 (15.6) | 8 (10.7) | 4  (8.5) | 4  (8.3) | 30 (20.3) | 18 (11.5) |  |
| **Serious TEAE** | 50 (50.5) | 42 (42.9) | 51 (53.1) | 35 (33.0) | 60 (50.8) | 49 (38.0) | 41 (53.2) | 28 (37.3) | 26 (55.3) | 25 (52.1) | 75 (50.7) | 52 (33.3) |  |
| **TEAE Leading to Dose Modification in Any Study Treatment** | 87 (87.9) | 71 (72.4) | 86 (89.6) | 85 (80.2) | 105 (89.0) | 99 (76.7) | 68 (88.3) | 57 (76.0) | 43 (91.5) | 39 (81.3) | 130 (87.8) | 117 (75.0) |  |
| **TEAE Leading to Dose Reduction in Any Study Treatment** | 73 (73.7) | 52 (53.1) | 68 (70.8) | 52 (49.1) | 81 (68.6) | 65 (50.4) | 60 (77.9) | 39 (52.0) | 35 (74.5) | 29 (60.4) | 106 (71.6) | 75 (48.1) |  |
| **TEAE Leading to Dose Interruption in Any Study Treatment** | 85 (85.9) | 60 (61.2) | 82 (85.4) | 79 (74.5) | 102 (86.4) | 88 (68.2) | 65 (84.4) | 51 (68.0) | 42 (89.4) | 36 (75.0) | 125 (84.5) | 103 (66.0) |  |
| **TEAE Leading to Study Treatment Discontinuation** | 22 (22.2) | 16 (16.3) | 19 (19.8) | 16 (15.1) | 24 (20.3) | 20 (15.5) | 17 (22.1) | 12 (16.0) | 15 (31.9) | 10 (20.8) | 26 (17.6) | 22 (14.1) |  |
| **TEAE Leading to Death** | 7  (7.1) | 6  (6.1) | 5  (5.2) | 5  (4.7) | 8  (6.8) | 7  (5.4) | 4  (5.2) | 4  (5.3) | 0 | 4  (8.3) | 12  (8.1) | 7  (4.5) |  |
|  | **IMiD refractory** | | **Prior bortezomib only** **as induction for ASCT** | | **ASCT** | | **No ASCT** | |  | | | | |
|  | **XVd**  **n= 74**  **n (%)** | **Vd**  **n= 85**  **n (%)** | **XVd**  **n=37**  **n (%)** | **Vd**  **n= 28**  **n (%)** | **XVd**  **n= 76**  **n (%)** | **Vd**  **n= 61**  **n (%)** | **XVd**  **n= 119**  **n (%)** | **Vd**  **n= 143**  **n (%)** |  |  |  |  |  |
| **Treatment-Emergent Adverse Event** | 73 (98.6) | 82 (96.5) | 37  (100.0) | 28 (100.0) | 76 (100.0) | 60 (98.4) | 118 (99.2) | 138 (96.5) |  |  |  |  |  |
| **Grade 3/4 TEAE** | 61 (82.4) | 52 (61.2) | 30 (81.1) | 14 (50.0) | 64 (84.2) | 35 (57.4) | 90 (75.6) | 79 (55.2) |  |  |  |  |  |
| **Grade 4 TEAE** | 11 (14.9) | 10 (11.8) | 14 (37.8) | 5  (17.9) | 20 (26.3) | 9 (14.8) | 14 (11.8) | 13  (9.1) |  |  |  |  |  |
| **Serious TEAE** | 41 (55.4) | 32 (37.6) | 17 (45.9) | 9  (32.1) | 41 (53.9) | 21 (34.4) | 60 (50.4) | 56 (39.2) |  |  |  |  |  |
| **TEAE Leading to Dose Modification in Any Study Treatment** | 64 (86.5) | 64 (75.3) | 32 (86.5) | 23 (82.1) | 68 (89.5) | 50 (82.0) | 105 (88.2) | 106 (74.1) |  |  |  |  |  |
| **TEAE Leading to Dose Reduction in Any Study Treatment** | 53 (71.6) | 40 (47.1) | 25 (67.6) | 17 (60.7) | 59 (77.6) | 36 (59.0) | 82 (68.9) | 68 (47.6) |  |  |  |  |  |
| **TEAE Leading to Dose Interruption in Any Study Treatment** | 60 (81.1) | 57 (67.1) | 31 (83.8) | 21 (75.0) | 67 (88.2) | 47 (77.0) | 100 (84.0) | 92 (64.3) |  |  |  |  |  |
| **TEAE Leading to Study Treatment Discontinuation** | 15 (20.3) | 10 (11.8) | 7  (18.9) | 1  (3.6) | 19 (25.0) | 11 (18.0) | 22 (18.5) | 21 (14.7) |  |  |  |  |  |
| **TEAE Leading to Death** | 4  (5.4) | 5  (5.9) | 2  (5.4) | 0 | 3  (3.9) | 1  (1.6) | 9  (7.6) | 10  (7.0) |  |  |  |  |  |

ASCT, autologous stem cell transplant; IMiD, immunomodulatory drug; PI, proteasome inhibitor; TEAE, treatment-emergent adverse event

**Figure S1**

**
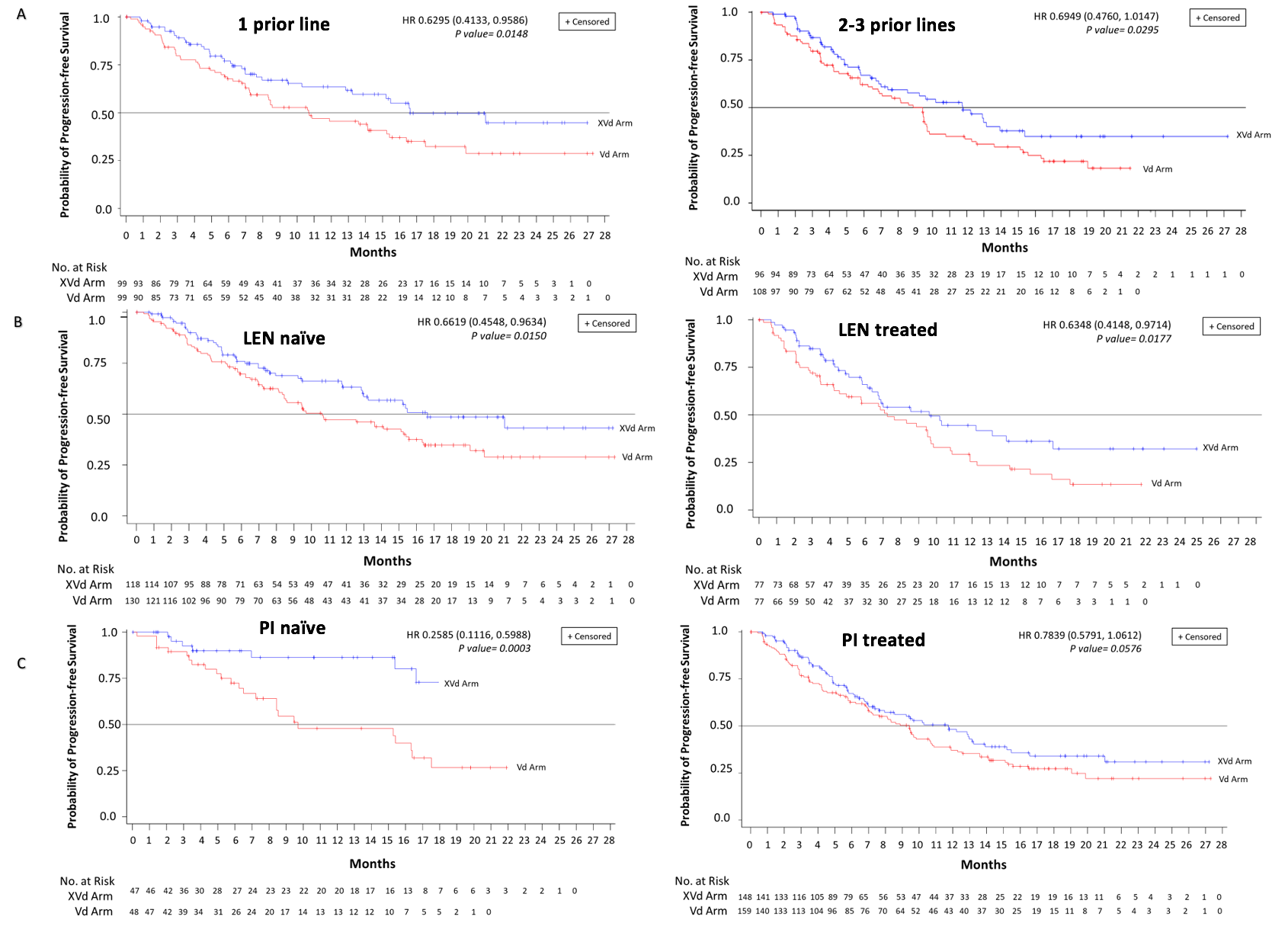
**

**
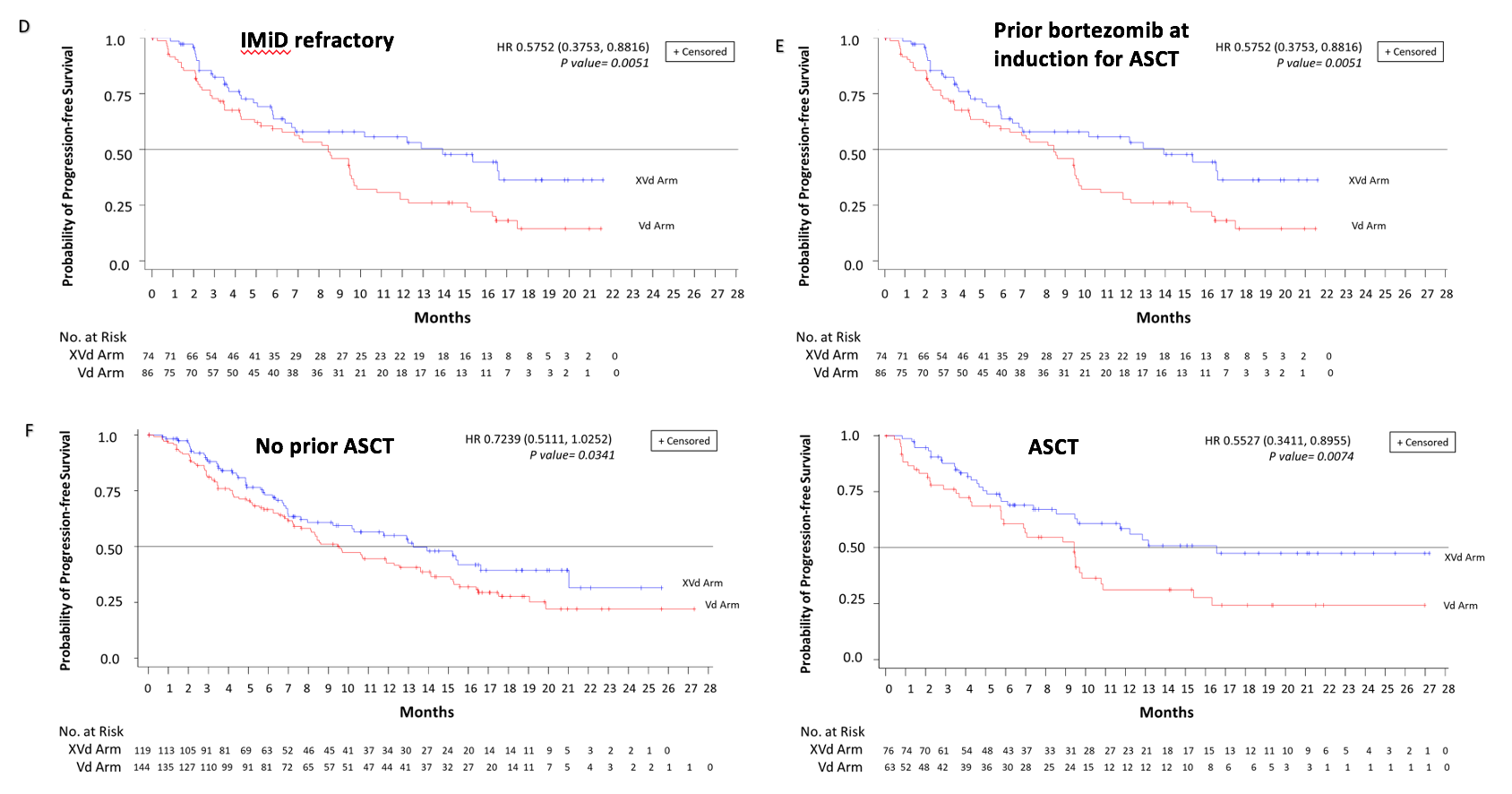
**

**Figure S1. Progression-free survival.** Kaplan-Meier curves for progression-free survival in patients **A**) that received one prior line (left) or 2-3 prior lines of therapy (right); **B)** lenalidomide naïve (left) and lenalidomide treated (right) **C)** PI naïve (left) and PI treated (right); **D)** IMiD refractory; **E)** prior bortezomib only as induction for ASCT; and **F)** prior ASCT (left) and no prior ASCT (right). ASCT, autologous stem cell transplant; IMiD, immunomodulatory drug; PI, proteasome inhibitor
